# Promotion of embryonic cortico-cerebral neuronogenesis by miR-124

**DOI:** 10.1186/1749-8104-4-40

**Published:** 2009-11-02

**Authors:** Nicola Antonio Maiorano, Antonello Mallamaci

**Affiliations:** 1 International School for Advanced Studies (SISSA/ISAS), Trieste, Italy

## Abstract

**Background:**

Glutamatergic neurons of the murine cerebral cortex are generated within periventricular proliferative layers of the embryonic pallium, directly from apical precursors or indirectly via their basal progenies. Cortical neuronogenesis is the result of different morphogenetic subroutines, including precursor proliferation and death, changes in histogenetic potencies, and post-mitotic neuronal differentiation. Control of these processes is extremely complex, involving numerous polypeptide-encoding genes. Moreover, many so-called 'non-coding genes' are also expressed in the developing cortex. Currently, their implication in corticogenesis is the subject of intensive functional studies. A subset of them encodes microRNAs (miRNAs), a class of small RNAs with complex biogenesis that regulate gene expression at multiple levels and modulate histogenetic progression and are implicated in refinement of positional information. Among the cortical miRNAs, miR-124 has been consistently shown to promote neuronogenesis progression in a variety of experimental contexts. Some aspects of its activity, however, are still controversial, and some have to be clarified. An in depth *in vivo *characterization of its function in the embryonic mammalian cortex is still missing.

**Results:**

By integrating locked nucleic acid (LNA)-oligo *in situ *hybridization, electroporation of stage-specific reporters and immunofluorescence, we reconstructed the cortico-cerebral miR-124 expression pattern during direct neuronogenesis from apical precursors and indirect neuronogenesis via basal progenitors. The miR-124 expression profile in the developing embryonic cortex includes an abrupt upregulation in apical precursors undergoing direct neuronogenesis as well as a two-step upregulation in basal progenitors during indirect neuronogenesis. Differential post-transcriptional processing seems to contribute to this pattern. Moreover, we investigated the role of miR-124 in embryonic corticogenesis by gain-of-function approaches, both *in vitro*, by lentivirus-based gene transfer, and *in vivo*, by *in utero *electroporation. Following overexpression of miR-124, both direct neuronogenesis and progression of neural precursors from the apical to the basal compartment were stimulated.

**Conclusion:**

We show that miR-124 expression is progressively up-regulated in the mouse embryonic neocortex during the apical to basal transition of neural precursor cells and upon their exit from cell cycle, and that miR-124 is involved in the fine regulation of these processes.

## Background

The glutamatergic neuronal complement of the mouse cerebral cortex is generated from neural precursors within periventricular proliferative layers of the embryonic pallium from embryonic day 11 (E11) onward [1,2]. Neural precursors include apical elements undergoing interkinetic nuclear migration (self-renewing neural stem cells and neuronally committed short neural precursors, also termed 'pin-like cells') as well as basal elements dividing far from the ventricle (neuronally committed intermediate precursor cells) [3-7]. Neurons originate from apical precursors directly or via their intermediate precursor cell progenies [8-10]. Throughout cortical development, indirect neuronogenesis is usually much more frequent than direct neuronogenesis [11].

Kinetics of neuronal generation emerges as a result of different basic morphogenetic subroutines, such as precursor proliferation and death, transitions among distinct proliferative compartments, cell cycle exit, and post-mitotic neuronal differentiation. Control of these subroutines is extremely complex, involving a large number of polypeptide-encoding genes belonging to distinct structural and functional families [9,12-19]. In addition to polypeptide-encoding mRNAs, a huge number of so-called non-coding RNAs are expressed in the developing central nervous system (CNS). Their expression patterns and their functions are presently the subject of intense investigation [20-22].

MicroRNAs (miRNAs) are a class of small non-coding RNA that are mainly transcribed by either RNA polymerase II or III as long precursors, processed by the sequential activities of the RNAse III enzymes Drosha and Dicer and eventually incorporated into bioactive RISC complexes [23,24]. miRNA functions include promotion of mRNA degradation and sequestration as well as inhibition of mRNA translation [25]. A huge number of miRNAs are specifically expressed in the developing embryo, where they are implicated in regulating histogenetic progression [26-29] as well as in refining positional information [30].

Among the best characterized miRNAs specifically expressed in the CNS is miR-124 [31]. Its expression goes up during neuronal differention, both prenatal and post-natal [32,33]. miR-124 over-expression channels non-neural HeLa cells to neuron-specific molecular profiles [34], inhibits proliferation in medulloblastomas and adult neural precursors [35,36] and promotes neuronal differentiation of committed neural precursors [36,37]. The molecular mechanisms underlying its action have been the subject of intensive investigation and include stimulation of neuron-specific transcriptome splicing [38], cross-talk with the general anti-neuronal REST/SCP1 transcriptional machinery [39,40], modulation of neuron-specific chromatin remodeling [41], down-regulation of the neuronogenesis-inhibitor *Sox9 *[36], and modulation of β1-integrin-dependent attachment of neural stem cells to the basal membrane [42]. So far, however, the role of miR-124 in mammalian embryonic corticogenesis has been determined *in vivo *only partially. Makeyev *et al *[38] performed cross-correlation studies on the expression of miR-124 and selected targets of it. Both Makeyev *et al *[38] and De Pietri-Tonelli *et al *[43] analyzed the consequences of cortico-cerebral ablation of the whole miR machinery following conditional *Dicer *knock-out. A reduction in proliferation has recently been reported to occur in the mammalian embryonic spinal cord upon combined miR-9*/miR-124 overexpression in neural precursors [41]. miR-124 functions have also been studied *in vivo *in the developing chick spinal cord [40,42], however, these studies led to some contrasting conclusions.

By integrated use of *in utero *electroporation of stage-specific reporter genes, locked nucleic acid (LNA)-oligo *in situ *hybridization and immunofluorescence, we investigated miR-124 expression in the developing mouse cortex. Then, by *in vitro *lentivirus-based gene transfer and *in utero *electroporation of miR-124-expressing plasmids, we addressed the roles played by this molecule in the regulation of embryonic cortico-cerebral neuronogenesis.

## Results

### Expression pattern

**Figure 1:**
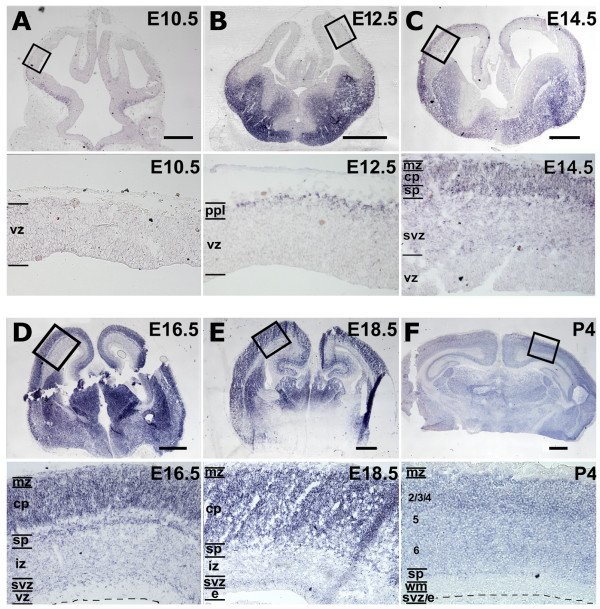
**Time course analysis of miR-124 expression**. **(A-F) ***In situ *hybridization of miR-124 on midfrontal E10.5 to post-natal day 4 (P4) mouse telencephalic sections. Magnifications of boxed areas are shown below each panel. Scale bars = 400 μm. Abbreviations: cp, cortical plate; e, ependyma; iz, intermediate zone; mz, marginal zone; ppl, preplate; sp, subplate; svz, subventricular zone; vz, ventricular zone; wm, white matter; 2/3/4, 5 and 6 are cortical layers.

We systematically studied the miR-124 expression pattern in the developing mouse cerebral cortex by LNA-oligo *in situ *hybridization [44]. No miR-124 signal was detectable in the cortex at E10.5, although it was strongly expressed by post-mitotic neurons of the ganglionic eminence at this time (Figure 1A). At E12.5, a light and diffuse signal was found throughout the cortical ventricular zone (VZ) and a stronger one within postmitotic neurons of the cortical preplate (Figure 1B). The pattern became more complex at E14.5, when three distinct radial expression domains could be distinguished. A faint signal was still detectable within the VZ, an intermediate signal appeared in the subventricular zone (SVZ), and a strong signal demarcated presumptive subplate, cortical plate and marginal zone. Remarkably, a few intensely labeled cells could be seen at the VZ-SVZ border (Figure 1C). This profile was basically retained at E16.5 and E18.5. At both these ages, miR-124 staining could distinguish the subplate from the cortical plate. Moreover, heavily labeled cells at the VZ-SVZ border were much more common. Finally, scattered cells expressing miR-124 at high levels also appeared in the VZ (Figure 1D, E). The miR-124 expression pattern became simplified at post-natal day 4 (P4), when the signal was restricted to the grey matter and was more intense in recently generated layer 2 to 4 neurons (Figure 1F). Remarkably, at all ages investigated, miR-124 expression was tightly restricted to the CNS, being completely absent in the surrounding mesenchymal tissue (Figure 1; Additional file 1) [45,46].

**Figure 2:**
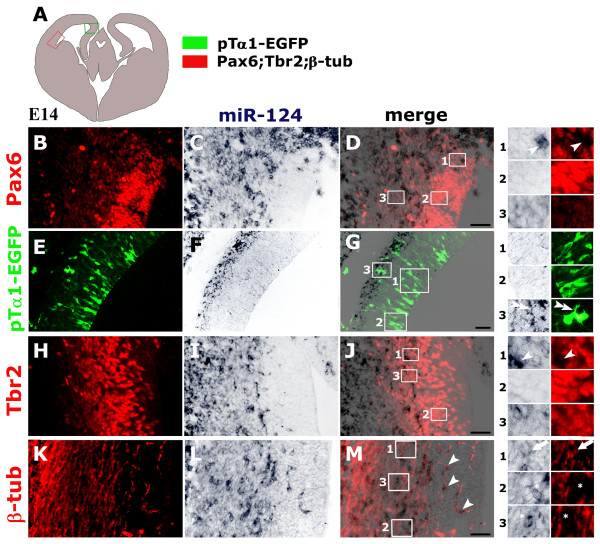
**Comparative profiling of cortical periventricular layers for miR-124 and markers of apical progenitors (Pax6 and pTα1-driven EGFP), basal progenitors (Tbr2) and post-mitotic neurons (β-tubulin)**. **(A) **Schematic of an E14 midfrontal telencephalic section showing the areas analyzed. **(B, E, H, K) **Immunofluorescence of Pax6, pTα1-driven EGFP, Tbr2 and neuron-specific β-tubulin, respectively. **(C, F, I, L) ***In situ *hybridization of miR-124. **(D, G, J, M) **Electronic merging of (B, C), (E, F) (H, I) and (K, K), respectively. Numbered magnifications of boxed areas in (D, G, J, M) show Pax6, pTα1-EGFP, Tbr2 and β-tubulin in cells expressing different levels of miR-124 Arrowheads in (D1), (J1) and (M) point to Pax6^-^/miR-124^high^, Tbr2^-^/miR-124^high ^and β-tubulin^-^/miR-124^high ^elements, respectively. Double arrowheads in (G3) denote pTα1-EGFP^+^/miR-124^high ^cells that no longer contact the ventricular cavity. Arrows in (M1) indicate SVZ β-tubulin^+ ^cells expressing intermediate levels of miR-124. Asterisks in (M2, M3) demarcate SVZ β-tubulin^- ^cells expressing high levels of miR-124. Scale bars = 100 μm.

Periventricular neural precursors fall into two distinct compartments: an apical, self-renewing compartment that includes cells with interkinetic nuclear migration and lying entirely within the VZ; and a basal compartment derived from the former, including non-motile cells which lie in both the VZ and SVZ [8-10]. To finely map the previously described different miR-124 expression levels to these compartments, these levels were compared with the distribution of specific protein markers at E14.5. Apical precursors, characterized by high Pax6 expression [7], generally displayed faint miR-124 staining (Figure 2D, box 2). This also specifically applies to a subset of these precursors - the short neural precursors [4] or 'pin-like cells' [5] - that are committed to neuronogenesis and distinguishable at E12.5 by *in utero *electroporation using enhanced green fluorescent protein (EGFP) immunoreactivity driven by the tubulin α1 promoter [4] and by the retention of a process connecting them with the ventricle (Figure 2G, boxes 1 and 2). Heterogeneous miR-124 expression was conversely detectable in basal precursors, characterized by weak Pax6 staining and robust Tbr2 immunoreactivity (Figure 2J) [7]. Among Tbr2^+ ^cells, presumptively younger elements, lying at more ventricular levels, showed weak miR-124 staining, like apical precursors (Figure 2J, box 2). On other hand, older elements, lying more marginally (and including basal progenitors as well as newborn neurons), displayed enhanced, intermediate miR-124 staining (Figure 2J, box 3). Furthermore, isolated Pax6^- ^and Tbr2^- ^cells expressed miR-124 at the highest level (Figure 2D, J, arrowheads). Their positions corresponded to those of VZ and SVZ cells, respectively, which co-expressed β-tubulin and abundant miR-124 (Figure 2M, arrowheads); they were thus considered to be newborn neurons. Remarkably, high miR-124 staining was not tightly restricted to post-mitotic neurons, but could also be detected in some β-tubulin^- ^elements within the SVZ (Figure 2M, asterisks); these were considered to be basal progenitors [47]. On the other hand, not all β-tubulin^+ ^neurons in the outer SVZ expressed miR-124 at the highest levels (Figure 2M, arrows), suggesting a remarkable variability in miR-124 upregulation along the neuronogenic lineage.

### Overexpression of miR-124

**Figure 3:**
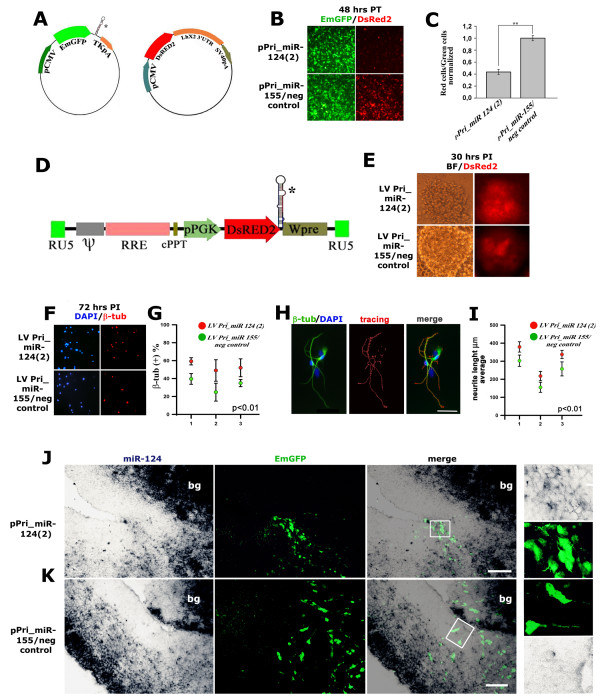
**Overexpression of miR-124 *in vitro *and *in vivo***. **(A) **Backbone of the expression plasmids pPri-miR-124(2) and pPri-miR-155/neg_control; miR-124-responsive sensor plasmid pCMV-DsRed2/*Lhx2*_3'UTR. The asterisk indicates the position of Pri-miR cDNA fragments. **(B, C) **Specific attenuation of DsRed2 expression in HeLa cells cotransfected with pPri-miR-124(2) and pCMV-DsRed2/*Lhx2*_3'UTR. PT, post-transfection. **(D) **Backbone of lentivectors LV_Pri-miR-124(2) and LV_Pri-miR-155/neg_control. The asterisk indicates the position of Pri-miR cDNA fragments. **(E) **DsRed2 expression in E12.5 primary cortico-cerebral progenitors infected by lentiviruses LV_Pri-miR-124(2) and LV_Pri-miR-155/neg_control at a multiplicity of infection of 40 and kept for 30 h in DMEM:F12:N2 medium supplemented with 2.5% fetal calf serum. PI, post-infection. BF, bright field. **(F, G) **Differential β-tubulin immunoprofiling of acutely infected, E12.5 dissociated cortical progenitor cells, at 72 h after infection. **(H) **Example of neurite outgrowth evaluation by immunostaining and subsequent NeuriteTracer^® ^analysis. **(I) **Differential neurite outgrowth in low density cortical progenitor cells 72 h after infection at E12.5, calculated for three different experiments by NeuriteTracer^®^. **(J, K) ***In vivo *overexpression of miR-124 in lateral neocortex. Distribution of miR-124 and pCMV-driven EmGFP on E14.5 midfrontal telencephalic sections from brains electroporated at E12.5 with the plasmids pPri-miR-124(2) and pPri-miR-155/neg_control, respectively. Magnifications of boxed insets of electronic merges are shown to the right. Abbreviations: bg, basal ganglia. In (C) error bars represent the standard error of the mean calculated among the means of each experiment; **P < 0.01, as evaluated by ANOVA test; N = 3+3. In (G, I) error bars represent the standard error of the mean calculated within each experiment; the *P *< 0.01 value was evaluated by *t*-test (one-tail, paired); N = 3+3. Scale bars = 40 μm (H) and 100 μm (I).

To cast light on the role played by miR-124, we developed a set of molecular tools for gain-of-function analysis. We cloned the Pri-miR-124(2) cDNA fragment [48,49] into the BLOCK-iT™ expression vector (Invitrogen) in-between the pCMV-EmGFP and TKpA modules in place of Pri-miR-155 derivative cDNA sequences (plasmid pPri-miR-124(2)). BLOCK-iT was used as a negative control expression vector (pPri-miR-155neg_control). To assess the effectiveness of pPri-miR-124(2) to over-express mature miR-124, we built up a dedicated sensor plasmid, cloning a *Lhx2*_3' untranslated region cDNA fragment harboring two miR-124 responsive elements [48-50] into the pDsRed2-N1 plasmid (Clontech) in-between the pCMV-DsRed2 and SV40pA modules (plasmid pmiR-124-sensor) (Figure 3A). Compared with pPri-miR-155neg_control/pmiR-124-sensor control, cotransfection of pPri-miR-124(2) and pmiR-124-sensor in HeLa cells specifically reduced the fraction of fluorescent cells expressing DsRed2 by about 60% (Figure 3B, C). To overexpress miR-124 in primary cortical precursor cells, we transferred the Pri-miR-124(2) cDNA fragment into the DsRed2 derivative of the constitutive lentiviral expressor pCCLsin.PPT.prom.EGFP.Wpre [51]. Transduction of primary cortical precursor cells with the resulting LV_Pri-miR-124(2) promoted neuronal generation, as shown by the increase in β-tubulin-expressing cells at 72 hours post-infection (Figure 3F, G). Enhancement of neuronal differentiation induced by LV_Pri-miR-124(2) was further confirmed by the increase in average total neurite length, calculated at 72 hours post-infection on low density cultures by NeuriteTracer^® ^(Figure 3H, I) [52]. Remarkably, these effects took place specifically when using 2.5% fetal calf serum (FCS) (Figure 3F-I). *In vivo *electroporation of pPri-miR-124(2) into the E12.5 lateral cortex resulted in specific upregulation of miR-124 in periventricular layers (Figure 3J, K). However, a more detailed analysis showed that the amplitude of this upregulation was moderate and only strongly electroporated periventricular cells expressed miR-124 at levels above the local natural range (Additional file 2). This might depend on poor processing of Pri-miR sequences to mature miRNAs that is peculiar to undifferentiated cells [53-55]. Consistent with this hypothesis, neuroblasts infected by LV_Pri-miR-124(2) and allowed to differentiate under FCS specifically and progressively down-regulated DsRed2 (Additional file 3) concurrent with enhanced maturation of their primary DsRed2/miR-124(2) transcripts to Pre-miR-124.

### *In vivo *promotion of neuronogenesis by miR-124

**Figure 4:**
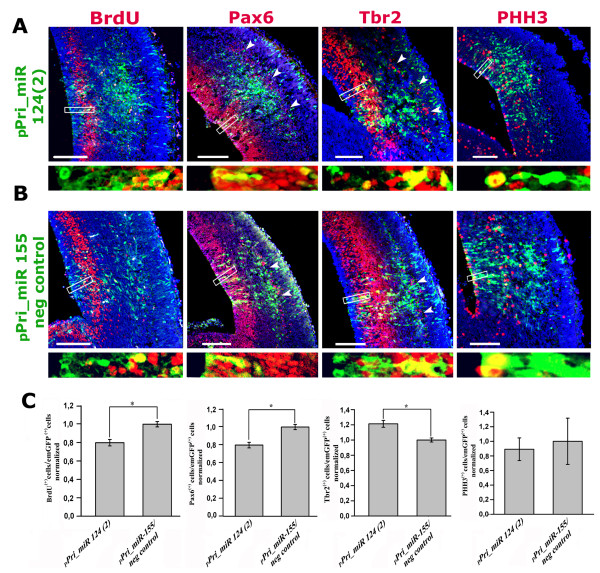
**Immunoprofiling of cortical periventricular layers after *in utero *electroporation of plasmids pPri-miR-124(2) and pPri-miR-155/neg_control; part I**. **(A, B) **Distribution of terminally administered BrdU, Pax6, Tbr2, phospho-histone3 (PHH3) and pCMV-driven EmGFP on E14.5 mid-frontal sections from brains electroporated *in utero *at E12.5 with plasmids pPri-miR-124(2) and pPri-miR-155/neg_control. Arrowheads point to abventricular displaced cells expressing Pax6 and Tbr2. Insets illustrate examples of EGFP/marker co-localizations, which were the subject of counting and statistical analyses. **(C) **Fractions of EmGFP^+ ^cells immunoreactive for BrdU, Pax6, Tbr2 and PHH3. *P < 0.05, as calculated by ANOVA test; N = 5+5 for BdU and Tbr2; N = 4+4 for Pax6; N = 3+3 for PHH3. Scale bars = 100 μm.

**Figure 5:**
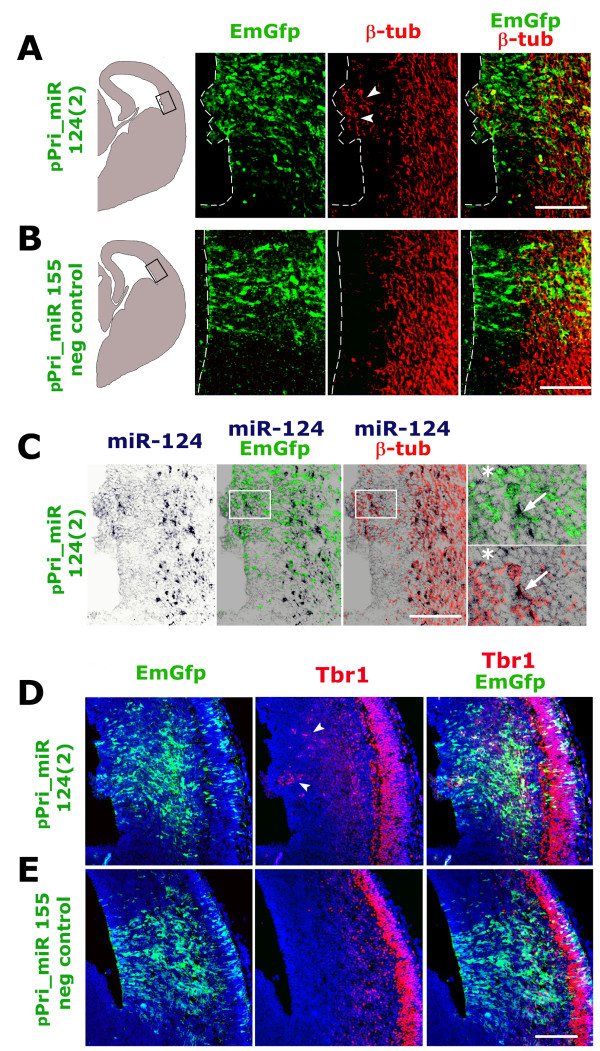
**Immunoprofiling of cortical periventricular layers after *in utero *electroporation of plasmids pPri-miR-124(2) and pPri-miR-155/neg_control; part II**. **(A, B, D, E) **Distribution of β-tubulin, Tbr1 and pCMV-driven EmGFP on E14.5 mid-frontal sections from brains electroporated *in utero *at E12.5 with plasmids pPri-miR-124(2) and pPri-miR-155/neg_control. In (A, D), arrowheads point to periventricular cells expressing β-tubulin and Tbr1, respectively. **(C) **Comparison of pCMV-driven EmGFP and neuron-specific β-tubulin with miR-124 expression in the electroporated area shown in (A, B, D, E). Magnifications of boxed insets to the right show an EmGFP^+ ^electroporated cell co-expressing huge amounts of miR-124 and β-tubulin (arrow), as well as another EmGFP^+^/miR-124^+ ^cell negative for β-tubulin (asterisk). Electroporated zones shown throughout Figure 5 correspond to the boxed areas in the schematics of (A, B). Scale bars = 100 μm.

**Figure 6:**
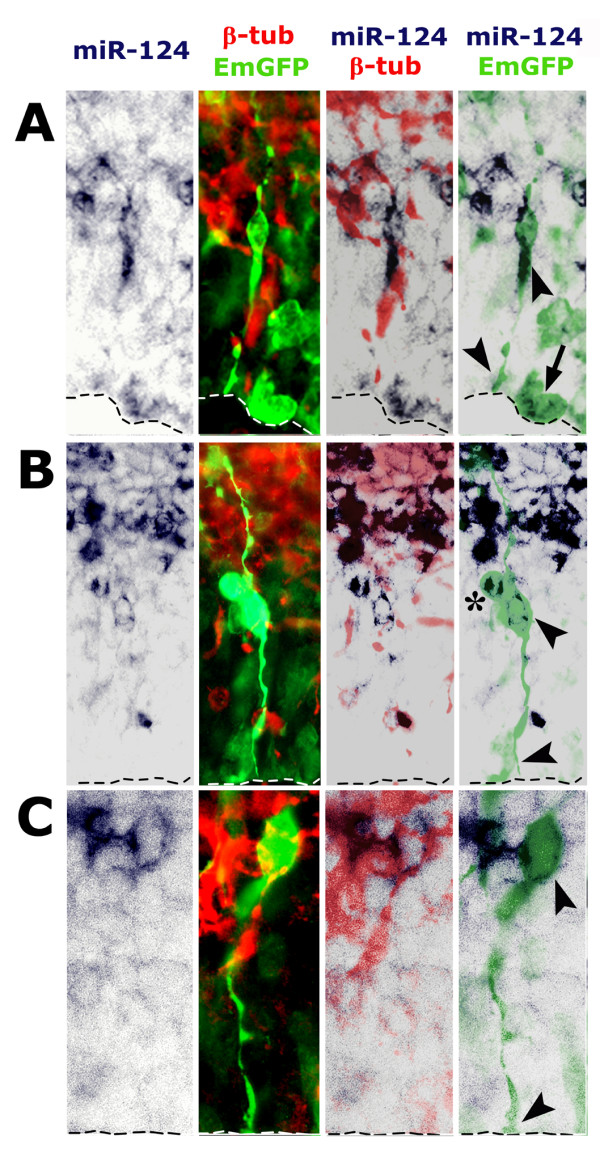
**Specific miR-124 overexpression in pPri-miR-124(2) electroporated periventricular neural precursors of the E14.5 cortex**. Distribution of miR-124, pCMV-driven EmGFP and neuron-specific β-tubulin in mid-frontal sections from brains electroporated *in utero *at E12.5 with pPri-miR-124(2). miR-124 may be specifically detected in: apical progenitors still connected to the ventricle (arrowheads in (A, B)) or undergoing mitosis (arrow in (A)); basal progenitors (asterisk in (B)); and nascent neurons still connected to the ventricle (arrowheads in (C)).

The miR-124 expression pattern reported above suggested active involvement in promotion of cortico-cerebral neuronogenesis. To confirm this and to clarify the cellular mechanisms underlying such promotion, we electroporated pPri-miR-124(2) or pPri-miR-155neg_control into the lateral ventricle of E12.5 mouse embryos and, 2 days later, immunoprofiled emerald green fluorescent protein (EmGFP)^+ ^electroporated cells and their progenies for molecular markers of neural precursors and newborn neurons. For each marker, at least three embryos electroporated with pPri-miR-124(2) and three electroporated with pPri-miR-155neg_control constructs (N ≥ 3+3) were analyzed and, for every embryo, at least 400 EmGFP^+ ^cells were scored. Upon pPri-miR-124(2) electroporation, the fraction of intermitotic EmGFP^+ ^cells was specifically reduced by 20% (N = 5+5; *P *< 0.05) when assessed by the S-phase marker terminally administered bromodeoxyuridine (BrdU). However, it did not display any statistically relevant change when evaluated by the M-phase marker phospho-histone3 (N = 3+3). EmGFP^+ ^cells expressing the apical precursor marker Pax6 decreased by 20% (N = 4+4; *P *< 0.05); conversely, those positive for the basal precursor marker Tbr2 increased by a similar percentage (N = 5+5; *P *< 0.05) (Figure 4). Electroporation of both pPri-miR-124(2) and pPri-miR-155neg_control also led to displacement of apical Pax6^+ ^and basal Tbr2^+ ^precursors towards the cortical plate (in six out of eight and eight out of ten embryos, respectively; Figure 4A, B, arrowheads). This phenomenon was not restricted to electroporated cells, but occurred mainly in their close surroundings. Since the two plasmids share a similar stem-and-loop Pri-miR moiety, to assess if such displacement were specifically linked to over-expression of this structure, we performed a further electroporation with the control plasmid pEGFP-C1™ (Clontech), which harbors the same pCMV-GFP module but is missing the Pri-miR element. Remarkably, even in this case, Pax6 and Tbr2 displacement was evident (three out of three electroporated embryos for both), ruling out that it was due to the Pri-miR moiety and indicating it was a consequence of the *in utero *electroporation protocol used (Additional file 4, arrowheads). In two out of five analyzed embryos, pPri-miR-124(2) electroporation specifically elicited strong activation of the post-mitotic markers β-tubulin (Figure 5A, B, arrowheads) and Tbr1 [7] (Figure 5D, E, arrowheads) as well as neurite outgrowth (Additional file 5, arrowheads) in the VZ. These effects were associated with strong over-expression of miR-124 in this zone (Figure 5C, arrows). In summary, overexpression of miR-124 seems to promote precursor transition from the apical to the basal compartment and to stimulate direct differentiation of apical progenitors to post-mitotic neurons. As suggested by activated-Caspase3 immunoprofiling of electroporated tissues (N = 4+4), such shifts seem not to arise as a consequence of differential cell death (Additional file 6). Finally, high-level miR-124 expression elicited in apical progenitors (including pin-like cells; Figure 6, arrowheads) upon *in vivo *electroporation may support these phenomena.

Looking for mechanisms linking miR-124 overexpression with promotion of apical-to-basal transition, we assayed expression of β1-integrin. This protein is necessary for integrity of adherens junctions among radial glial cells and the subpial basal membrane [56] and is an established target of miR-124 in chicken [42]. The cortical expression profile of β1-integrin normally includes a strong signal in the VZ, transitional field and marginal zone and a palisade-like pattern in the cortical plate, possibly corresponding to pial processes of radial glia and migrating neurons super-imposed on a weaker signal from resident neurons (Additional file 7). This domain is quite complementary to that expressing miR-124 at high levels. Unexpectedly, however, electroporation of Pri-miR-124(2) in the mouse cerebral cortex did not elicit any detectable down-regulation of β1-integrin. This may mean that miR-124-dependent regulation of β1-integrin is peculiar to the chicken neural tube and does not take place in the mammalian cortex. Alternatively, this may be due to a poor sensitivity of our immunodetection technique in discerning subtle changes in antigen concentration.

## Discussion

In this study, by integrating LNA-oligo *in situ *hybridization, electroporation of stage-specific reporters and immunofluorescence, we carefully reconstructed the miR-124 expression pattern in the developing mouse cerebral cortex. Moreover, by *in vitro *lentivirus-based gene transfer and *in utero *electroporation of gain-of-function plasmids, we investigated the activity of this molecule in the embryonic neuronogenic process.

**Figure 7:**
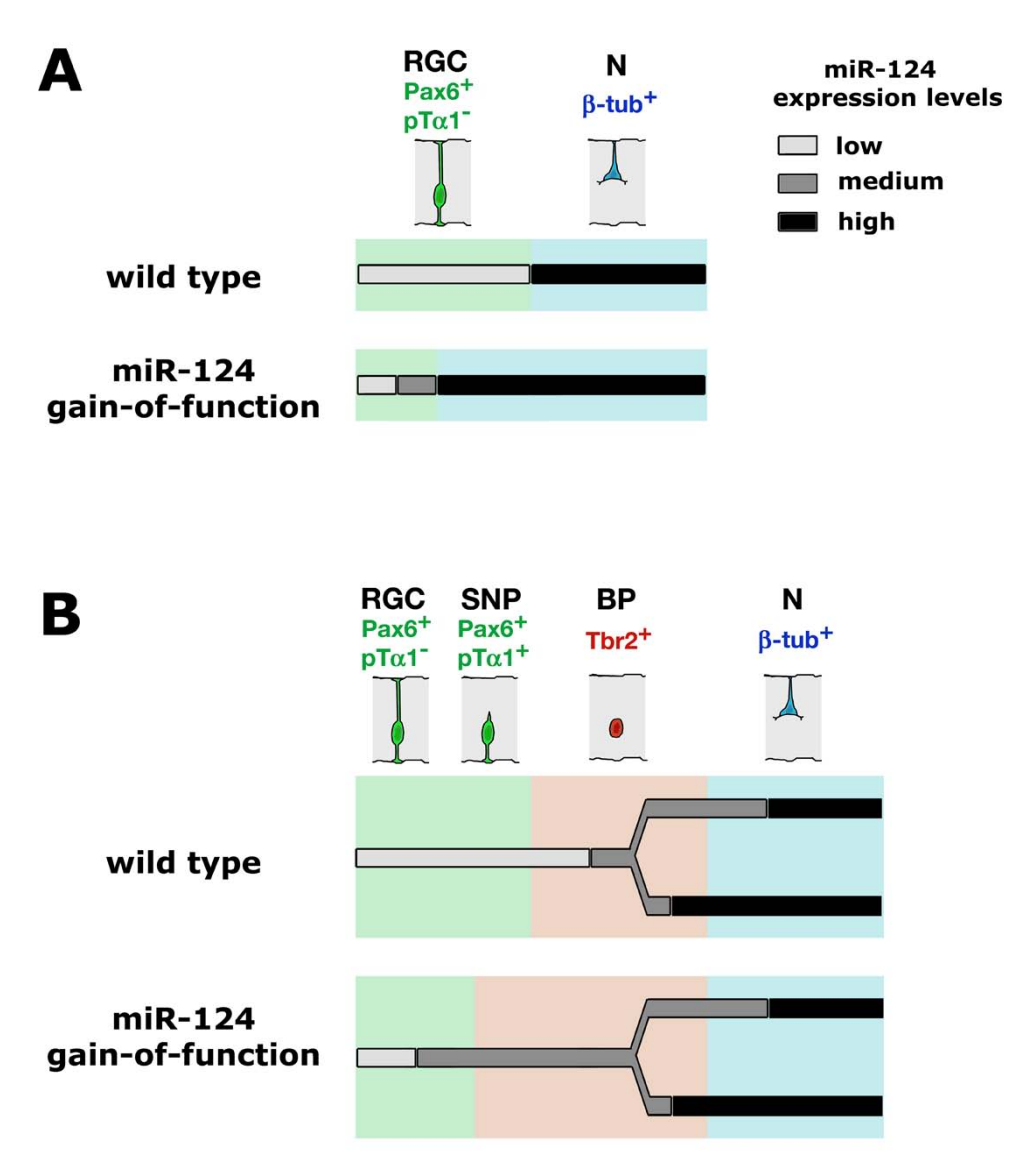
**Schematic of the miR-124 expression profile along neuronogenic lineages and the phenotype of miR-124 gain-of-function electroporated cortices**. **(A, B) **One-step and two-step changes in miR-124 expression levels during direct (A) and indirect (B) neuronogenesis. Stimulation of direct neuronogenesis and expansion of the basal compartment occurs at the expense of the apical one upon miR-124 over-expression. RGC, radial glial cell; SNP, short neural precursor; BP, basal progenitor; N, neuron.

We confirmed that miR-124 is progressively up-regulated during embryonic neuronogenesis, as previously reported [36,38,57,58]. In particular, with the appearance of the SVZ, we found that miR-124 displayed three distinct expression levels: low in the VZ [36,57], intermediate in the SVZ and high in more marginal layers (Figure 1; Additional file 1). The presence of isolated β-tubulin^+ ^cells and Pax6^- ^cells within the VZ, both of which expressed miR-124 highly, suggested that an abrupt upregulation of miR-124 might occur during direct neuronogenesis (Figures 2 and 7A). Conversely, the miR-124 expression profile appeared biphasic in indirect neuronogenesis; a transition from low to intermediate expression took place within Tbr2^+ ^basal progenitors that had lost contact with the ventricular cavity, and a further upregulation was localized in basal progenitors or early post-mitotic β-tubulin^+ ^neurons (Figures 2 and 7B). This scenario is reminiscent to what was found by Doetsch and colleagues in the adult SVZ [36]. In their study, a first upregulation of miR-124 occurred after the transition of neural stem cells to transit amplifying cells and a second upregulation was generally associated with the exit of neuroblasts from the cell cycle [36,56,59]. Finally, we specifically detected an accumulation of cells highly expressing miR-124 and with multipolar morphology at the border between the VZ and SVZ from E14.5 onward (Figures 1 and 2I, L). Such accumulation recalls the 'sojourn band' or 'multipolar accumulation zone', where newborn neurons settle before initiating their radial migration [1,60,61].

Electroporation of our gain-of-function constructs was followed by concerted upregulation of EmGFP and miR-124. Such upregulation was relatively weak for miR-124; however, levels of this miRNA in electroporated apical precursors were often above those of endogenously expressed miR-124 in the VZ, allowing functional perturbation of the system (Figures 3 and 6; Additional file 2). Limited production of miR-124 despite abundant levels of the EmGFP/Pri-miR-124(2) transcript available in apical progenitors might stem from suboptimal, regulated processing of this chimeric transcript to mature miRNA. This hypothesis is consistent with the discrepancy between the expression profiles of miR-124, which is mainly restricted to abventricular layers (Figures 1 and 2), and its precursors, which are conversely detectable at E14.5 at similar levels throughout the cortical wall [58]. This may also account for the progressive lowering of DsRed2 fluorescence we found in *in vitro *differentiating neurons harboring a DsRed2/Pri-miR-124(2) transgene. The idea that substantial modulation of miRNA levels may occur after transcription is not novel. In addition to transcriptional regulation [39,62], it has been suggested and experimentally proven that biogenesis of many miRNAs may be regulated at a variety of levels, including Drosha-dependent conversion of Pri-miR to Pre-miR [63], translocation of Pre-miR from the nucleus to the cytoplasm [64], Dicer-dependent conversion of Pre-miR to miR [55], and incorporation of miRNAs into RISC [65]. Modulation of Pri-miR processing is especially relevant to the proper regulation of neuro-specific and neuro-enriched miRNAs, including *let-7 *family members, *miR-128 *and *miR-138*, whose post-transcriptional maturation may dramatically increase with the transition from stem cells to post-mitotic differentiated elements [53-55]. Preferential confinement of the maturation of many miRNA precursors to late histogenesis is consistent with the integrity of stem cells within the cortical VZ of *Dicer *conditional-null mutants [38,43], as well as with the impaired differentiation abilities of *Dicer*^-/- ^embryonic stem cells [66]. Post-transcriptional regulation of miR-124 has already been addressed in the developing *Drosophila *nervous system, where dFMR1 is required for its proper biogenesis [67]. Further studies are required to clarify modulation of miR-124 expression in vertebrates.

By electroporating a Pri-miR-124(2) precursor into the developing mouse cortex, we were able to promote cortical neuronogenesis. We forced a fraction of ventricular precursors to leave the apical compartment and move to the basal compartment (Figure 4). We occasionally anticipated β-tubulin activation in pin-like cells (Figure 6C) and elicited an ectopic burst of neuronogenesis from apical progenitors within the VZ (Figure 5; Additional file 5). We replicated the last result *in vitro *by over-expressing Pri-miR-124(2) in dissociated cortical neuroblasts, but only when these precursors were kept under differentiating medium (Figure 3F-I). Inhibition of BrdU uptake and stimulation of direct neuronogenesis has been reported already in the chicken embryonic spinal cord, specifically upon electroporation of mature miR-124 [40,42]. A reduction in the number of dividing cells also takes place *in vivo *in the adult mouse SVZ upon Pri-miR-124(3) overexpression. Consistently, administration of antisense miR-124 to *in vitro *cultures of SVZ elements increases BrdU uptake by C-type transit amplifying cells and A-type neuroblasts, slowing down transition from the former to the latter [36]. Remarkably, we also found that miR-124 facilitates neuronogenesis in a permissive molecular environment, but is not able to initiate such a process *per se*, similar to what was previously described [37,38,68]. Finally, we did not find any increase in cell death upon Pri-miR-124(2) electroporation (Additional file 6), in contrast to what was previously reported for the chicken embryo [42]. This may be due to a variety of reasons, including differences between animal models, different CNS tracts studied, and different constructs used and electroporation protocols.

Lastly, by analyzing electroporated brains, we noticed a previously undescribed technical artifact. We detected a pronounced displacement of apical Pax6^+ ^and basal Tbr2^+ ^progenitors, just beneath the cortical plate, in both pPri-miR-124(2) and pPri-miR-155_neg_control electroporated brains (Figure 4A, B, arrowheads). This phenomenon was replicated upon electroporation of pEGFP-C1 (Additional file 4, arrowheads), which shares the pCMV-EGFP module with the above two plasmids but does not harbor the Pri-miR stem-loop moiety, indicating that miR-124 or stem-loop specificity are not involved in it. Displacement of apical and basal precursors took place only on the electroporated side, was mainly restricted to the middle of the electroporated zone, being undetectable in its surroundings, and was not cell-autonomous (Additional file 4). Despite the locality of the displacement, the electric field we applied was uniform throughout the E12.5 telencephalon, thanks to the 7 mm tweezer electrodes we used. This implies that this effect was not due to the electrical stress *per se*. Reasonably, it might originate from heavy metabolic loads weighing on electroporated precursors, possibly impairing the correct scaffold structure of the cortical wall. The mechanical damage induced by the injection needle might contribute to the priming of such an effect. Nevertheless, displacement of Pax6^+ ^and Tbr2^+ ^progenitors was equally present in controls and Pri-miR-124(2) electroporated embryos, and so does not affect the results of the miR-124 gain-of-function analysis.

## Conclusion

Our study makes two main observations. First, miR-124 is expressed in the developing embryonic cortex according to a complex pattern. It is upregulated sharply in apical precursors undergoing direct neuronogenesis and, via an intermediate expression level, in late basal progenitors during indirect neuronogenesis (Figure 7). Differential post-transcriptional processing seems to contribute to this pattern. Second, miR-124 overexpression stimulates direct neuronogenesis and promotes transition of neural precursors from the apical to the basal compartment (Figure 7).

These findings shed light on the role of miR-124 during early cortical development in mammals. Understanding the role of miRNAs during neurogenesis may be fundamental to uncovering the mechanisms that regulate the sizes of the different cell compartments in the CNS primordium.

## Materials and methods

### Animals and bromodeoxyuridine injection

Mice (*Mus musculus *strain CD1, purchased from Harlan-Italy Srl (San Pietro al Natisone, UD, Italy)) were maintained at the SISSA-CBM mouse facility and were staged by timed breeding and vaginal plug inspection. Animal handling and subsequent procedures were in accordance with European laws (European Communities Council Directive of November 24, 1986 (86/609/EEC)) and with National Institutes of Health guidelines. Embryos (E10.5 to E18.5) were harvested from pregnant dames killed by cervical dislocation. When required, BrdU was injected intraperitoneally into previously electroporated pregnant dams 45 minutes before the sacrifice at 150 μg/g bodyweight. Electroporated embryos were harvested immediately afterwards.

### Pri-miRNA and cDNA expression constructs

The pPri-miR-124(2) construct contains the 285-bp mouse Pri-miR-124(2) genomic fragment (chr3 (+):17695562-17695846) cloned into the BLOCK-iT™ expression vector (Invitrogen - Life Technologies Corporation, Carlsbad, CA, U.S.A.) in-between the pCMV-EmGFP and TK_pA modules using *Sal*I and *Xba*I enzyme restriction sites. pPri-miR-155neg_control contains the Pri-miR155 sequence in-between the pCMV-EmGFP and TK_pA modules (BLOCK-iT™, Invitrogen). The plasmid pmiR-124-sensor contains the 477-bp 3' untranslated region fragment of mouse *Lhx2 *(chr2 (+):38224759-38225235) cloned into the pDsRed2-N1 plasmid (Clontech Laboratories Inc., Mountain View, CA, U.S.A) in-between the pCMV-DsRed2 and SV40pA modules using *Not*I and *Eco*RV enzyme restriction sites. The pTα1-EGFP plasmid (a kind gift of E Ruthazer) harbors the GFP coding sequence under the control of the α-tubulin 1 promoter (pTα1). pLV_Pri-miR-124(2) and pLV_Pri-miR-155neg_control, encoding lentiviral RNA genomes, were generated as follows. Briefly, the Pri-miR-124(2) and Pri-miR-155neg_control *Dra*I/*Bgl*II fragments were transferred from pPri-miR-124(2) and pPri-miR-155neg_control, respectively, into the pDsRed2-N1 *Not*I-blunted/*Bgl*II-cut plasmid downstream of the DsRed2 module. Subsequently, the DsRed2-Pri-miR-124(2) and the Dsred2-Pri-miR-155neg_control *Age*I/*Sma*Ifragments were transferred from the resulting plasmids into the pCCLsin.PPT.prom.EGFP.Wpre [51]*Age*I/*Sal*I-blunted cut vector. pEGFP-C1 (Clontech) was used as control for *in utero *electroporation.

### Production and titration of lentiviral vectors

Plasmids pLV_Pri-miR-124(2) and pLV_Pri-miR-155neg_control were used to produce lentiviral vectors LV_Pri-miR-124(2) and LV_Pri-miR-155neg_control as previously described [51]. Titration of lentiviral vectors was performed by real-time PCR, as previously reported [69].

### miR-activity assay

HeLa cells grown in 10% FCS and Dulbecco's modified Eagle's medium with Glutamax (DMEM/Glutamax; Invitrogen) were co-transfected with either pPri-miR-124(2) or pPri-miR-155neg_control, each pre-mixed with pmiR-124-sensor plasmid at a molar ratio of 30:1, using Lipofectamine (Invitrogen) and according to the manufacturer's instructions. Forty-eight hours after transfection, photos of ten different randomly chosen fields of each plate were taken, using a Nikon Eclipse 80 i fluorescent microscope (20× lens) and a DS-2 MBWC digital microscope camera. Pictures were processed using Photoshop CS3 software and specific attenuation of DsRed2 signal was evaluated by comparing the numbers of single- and double-labeled cells. All cell counting was performed on coded samples, so that the experimenter was blind to the condition. The experiment was repeated three times and data analyzed using Excel 2008 and SigmaPlot.

### Lentiviral gene transduction on differentiating primary cortical precursor cells

To evaluate the fraction of β-tubulin^+ ^cells among differentiating primary cortical precursor cells, cerebral cortices of E12.5 embryonic brains were dissected as previously described [70]. Cells (3 × 10^6^) were plated onto each well of a 12-multiwell Falcon plate (Becton Dickinson, Franklin Lakes, NJ, U.S.A.) at a density of 10^3 ^cells/μl and cultured in DMEM/F12/Glutamax medium (Invitrogen) with N2 supplement (Invitrogen), 0.6% w/v glucose, 2 μg/ml heparin, 10 pg/ml fungizone and with or without 2.5% FCS. Cortical precursors were transduced with lentiviral vectors at a multiplicity of infection of 40. Medium was replaced 36 h post-transduction. For the experiments on neurite outgrowth, 5 × 10^5 ^cells were plated onto each well of a polylysined 12-multiwell plate (Falcon) at a density of 200 cells/μl and cultured as above. This lower density culture was necessary to allow for subsequent NeuriteTracer^® ^analysis of differentiating cells.

### Evaluation of neuronal frequencies *in vitro*

Seventy-two hours after lentiviral infection, *in vitro *transduced cells were dissociated with trypsin-EDTA for 5 minutes, left to attach on poly-L-lysine coated glass coverslips for 30 minutes and finally fixed in 4% paraformaldehyde. Staining was performed as previously described [71] with primary mouse monoclonal antibody anti-β-tubulin (1:300; clone Tuj1, Covance, Princeton, NJ, U.S.A.) and anti-mouse secondary antibody Alexa fluor 594 conjugates (1:500; Invitrogen). DAPI (4',6'-diamidino-2-phenylindole) was used as nuclear counterstaining. For each experiment 5 subject and 5 control fields were captured using a fluorescent Nikon Eclipse 80 i microscope (20× lens) and a DS-2 MBWC digital microscope camera. For each experiment, at least 300 subject and 300 control cells were counted. The experiment was repeated three times and data were analyzed as follows. Frequencies of β-tubulin^+ ^cells within each field were calculated. They were averaged for each experiment and each lentivirus; results (± standard error of the mean) are therefore plotted against experiment number. Finally, the 3 subject and the 3 control average frequencies obtained were analyzed by *t*-test (one-way, paired) and the *P*-value reported on the graph.

### Evaluation of *in vitro *neurite outgrowth

Seventy-two hours after lentiviral infection, *in vitro *transduced cells were fixed for 15 minutes in 4% paraformaldehyde. β-Tubulin/DAPI staining was performed as described in the Evaluation of neuronal frequencies *in vitro *section above, replacing the Alexa fluor 594 antibody with Alexa fluor 488. For each experiment 30 subject and 30 control fields were captured, using a fluorescent Nikon Eclipse 80 i microscope (40× lens) and a DS-2 MBWC digital microscope camera. For each experiment, at least 150 subject and 150 control β-tubulin^+ ^cells were sampled. Electronic files were imported into ImageJ and processed using the NeuriteTracer^® ^plugin according to the authors' instructions [52], and NeuriteTracer^® ^outputs - that is, average neurite lengths per neuron calculated per each field - were collected. The experiment was repeated three times and data were analyzed as follows. Average neurite lengths per neuron calculated for each field were averaged for each experiment and each lentivirus. The results (± standard error of the mean) were plotted against experiment number. Finally, the 3 subject and the 3 control average frequencies obtained were analyzed by *t*-test (one-tail, paired) and the *P*-value reported on the graph.

### *In utero *electroporation

Electroporation was carried out to transfect VZ cells *in utero *with mammalian expression vectors as described previously [4,72,73]. Briefly, uterine horns of E12.5 pregnant dams were exposed by midline laparotomy after anesthetization with ketamine (200 μg/g bodyweight) and xylazine (40 μg/g bodyweight). Then, 1.5 μl of a solution containing 3 μg of DNA plasmid mixed with 0.02% fast-green dye in phosphate buffered saline (PBS) was injected in the telencephalic vesiscle using a sharp pulled micropipette (hole external diameter about 30 μm) through the uterine wall and the amniotic sac. Platinum tweezer-style electrodes (7 mm diameter) were placed outside the uterus over the telencephalon and four pulses of 40 mV were applied (each 50 ms long; interval between consecutive pulses 950 ms) using a BTX ECM830 square wave pulse generator (Genetronics, San Diego, CA, U.S.A.). Electroporation was performed in about half of the embryos found in each uterine horn to avoid prolonged surgery time. The uterus was then replaced within the abdomen, the cavity was filled with warm sterile PBS, and the abdominal muscle and skin incisions were closed with silk sutures. Animals were left to recover in a warm clean cage. Harvesting of electroporated embryos was performed 2 days later, as described above.

### *In situ *microRNA hybridization

Brains from E10.5 to E18.5 embryos and 4-day-old CD1 mice (Harlan lab), as well as brain from E14.5 embryos electroporated 2 days earlier, were perfused with 4% paraformaldehyde overnight. Afterwards, brains were immersed in 30% sucrose (w/v) and embedded in OCT mounting medium. *In situ *hybridization was carried out on 10 μm coronal brain slices using miRCURY 5' DIG labeled detection probes (LNA) for mmu-miR-124, mmu-miR-425 and mmu-miR-207 according to the manufacturer's instructions (Exiqon, Vedbaek, Denmark), as previously described [44].

### Tissue immunofluorescence

Immunofluorescence analyses were performed as previously described [72]. Briefly, frozen sections were boiled in 10 mM sodium citrate, pH 6.0, and blocked in 10% fetal bovine serum and 0.1% Triton X-100 for 1 h at room temperature. Incubation with primary antibodies was performed at 4°C overnight. In the case of BrdU detection, epitopes were made accessible by HCl treatment, as previously described [75]. Secondary antibodies were applied to sections for 2 h at room temperature. The following primary antibodies were used: anti-β-tubulin mouse monoclonal (1:300; clone Tuj1, Covance, Princeton, NJ, U.S.A.), anti-Egfp chicken polyclonal (1:600; AbCam, Cambridge, MA, U.S.A.), anti phosphohistone-H3 rabbit polyclonal (1:400; Chemicon-Millipore, Billerica, MA, U.S.A.), anti-Tbr1 rabbit polyclonal (1:2,000; a gift from R Hevner, Seattle, USA), anti-Tbr2 rabbit polyclonal (1:600; AbCam), anti-active_caspase3 rabbit polyclonal (1:300; BD Biosciences Pharmingen, San Diego, CA, U.S.A.), anti-Pax6 rabbit polyclonal (1:500; AbCam), anti-β1-integrin rat monoclonal (1:500; clone VLA, Chemicon-Millipore, Billerica, MA, U.S.A.), and anti-BrdU mouse monoclonal (1:50; clone B44, BD Biosciences Pharmingen, San Diego, CA, U.S.A.). Secondary antibodies were conjugates of Alexa Fluor 488 and Alexa Fluor 594 (1:500; Invitrogen). DAPI was used as nuclear counterstaining. Finally, slices were washed and mounted in Vectashield Fluorescent Mounting Medium (Vector Labs, Burlingame, CA, U.S.A.). Immunofluorescence analyses on *in situ *hybridized coronal brain slices was performed as described above, after washing the LNA-hybridized sections for 1 h in PBS.

### Acquisition, processing and statistical analysis of *in vivo *immunoprofiling data

*In situ *hybridized sections with or without immunofluorescence analysis were imaged using a fluorescent Nikon Eclipse 80 i microscope and a DS-2 MBWC digital microscope camera. Such images were processed using Adobe Photoshop CS3 software.

For each marker under analysis, cell counting was performed on at least three different electroporated embryos for both pPri-miR-124(2) and pPri-miR-155 constructs (N ≥ 3+3); three sections from each electroporated embryo spaced 100 μm apart along the rostro-caual axis were inspected. In total, at least 400 EmGFP^+ ^cells per embryo were scored for double labeling, paying special attention to compare embryonic tissue electroporated at similar rostro-caudal and medio-lateral levels. Sections were photographed using a TCS SP2 Leica confocal microscope, generally collected as 5.0-μm-thick Z-stacks of 1,024 × 1,024 pixel images. Images were then imported into Photoshop CS3, where all cell countings were performed on coded samples, so that the experimenter was blind to the condition. Results were imported into Excel 2008, percentages of labeled cells were calculated for each brain and data relative to all brains electroporated with the same construct were averaged. Results are expressed as mean value ± standard error of the mean and were tested for statistical significance by one-way-ANOVA. Results shown are normalized against controls.

## Abbreviations

BrdU: bromodeoxyuridine; CNS: central nervous system; DMEM: Dulbecco's modified Eagle's medium; E: embryonic day; EGFP: enhanced green fluorescent protein; EmGFP: emerald green fluorescent protein; FCS: fetal calf serum; LNA: locked nucleic acid; miRNA: microRNA; PBS: phosphate buffered saline; SVZ: subventricular zone; VZ: ventricular zone.

## Competing interests

The authors declare that they have no competing interests.

## Authors' contributions

NAM and AM designed the study, NAM performed the experiments, and NAM and AM analyzed the data and wrote the manuscript. Both authors read and approved the final manuscript.

## Supplementary Material

Additional file 1 **Specific faint expression of miR-124 in the E14.5 VZ**. **(A-C) ***In situ *hybridization of miR-124 (A), miR-425 (B) and miR-207 (C) probes on mid-frontal E14.5 telencephalic sections. miR-425 and miR-207 are two miRNAs not expressed in the developing CNS [45,46]. Magnifications of boxed areas illustrate the faint staining detectable in the VZ (black arrowheads) but not in mesenchymal tissue (black harrow) upon miR-124 hybridization, as well as the absence of any signal in samples hybridized with miR-425 or miR-207 (asterisks). Scale bar = 100 μm. Abbreviations: cp, cortical plate; mes, mesenchymal tissue; svz, subventricular zone; vz, ventricular zone. Click here for file

Additional file 2 **Levels of miR-124 expression in the E14.5 VZ after *in vivo *E12.5 pPri-miR-124 electroporation**. Arrowheads in boxed inset magnifications denote mid-to-high miR-124 expression levels, which are specifically restricted to heavily electroporated elements. Scale bar = 100 μm. Click here for file

Additional file 3 **Time-course DsRed2 fluorescence in primary cortical precursor cultures infected with Pri-miR expressing lentiviruses**. Divergent temporal progression of DsRed2 fluorescence in E12.5 neuroblasts infected by LV_Pri-miR-124(2) or LV_Pri-miR-155/neg_control and allowed to differentiate in FCS. PI, post-infection. Click here for file

Additional file 4 **Displacement of apical, Pax6^+^, and basal, Tbr2^+^, precursors within the cortical wall of E14.5 brains electroporated 2 days earlier with pEGFP-C1**. Arrowheads point to abventricularly displaced Pax6^+ ^and Tbr2^+ ^elements, both positive and negative for electroporated EGFP. Such displaced cells were not detectable in the controlateral, non-electroporated side of same embryos (N = 3). Scale bar = 100 μm. Click here for file

Additional file 5 **VZ neuronal differentiation in E14.5 cerebral cortex electroporated 2 days earlier with pPri-miR-124(2)**. Arrowheads show outgrowing neurites within the VZ. Scale bar = 10 μm. Click here for file

Additional file 6 **Distribution of activated-Caspase3^+ ^apoptotic cells within the cortical wall of E14.5 brains electroporated 2 days earlier with pPri-miR-124(2) or pPri-miR-155/neg_control**. N = 4+4. Scale bar = 100 μm. Click here for file

Additional file 7 **Distribution of β1-integrin within the cortical wall of E14.5 brains electroporated 2 days earlier with pPri-miR-124(2) or pPri-miR-155/neg_control**. The arrowhead in (B) points to the pPri-miR-124(2)-electroporated region, which does not display any overt reduction of β1-integrin immunoreactivity. Arrows in (A-C) denote the cortical plate, where β1-integrin is down-regulated and restricted to radial glial fibers. Scale bar = 100 μm. Click here for file
