# Supplementary figures and images for: Promotion of embryonic cortico-cerebral neuronogenesis by miR-124
Source: Neural Dev. 2009 Nov 2;4:40. doi: 10.1186/1749-8104-4-40 (PMC2777883; doi:10.1186/1749-8104-4-40)

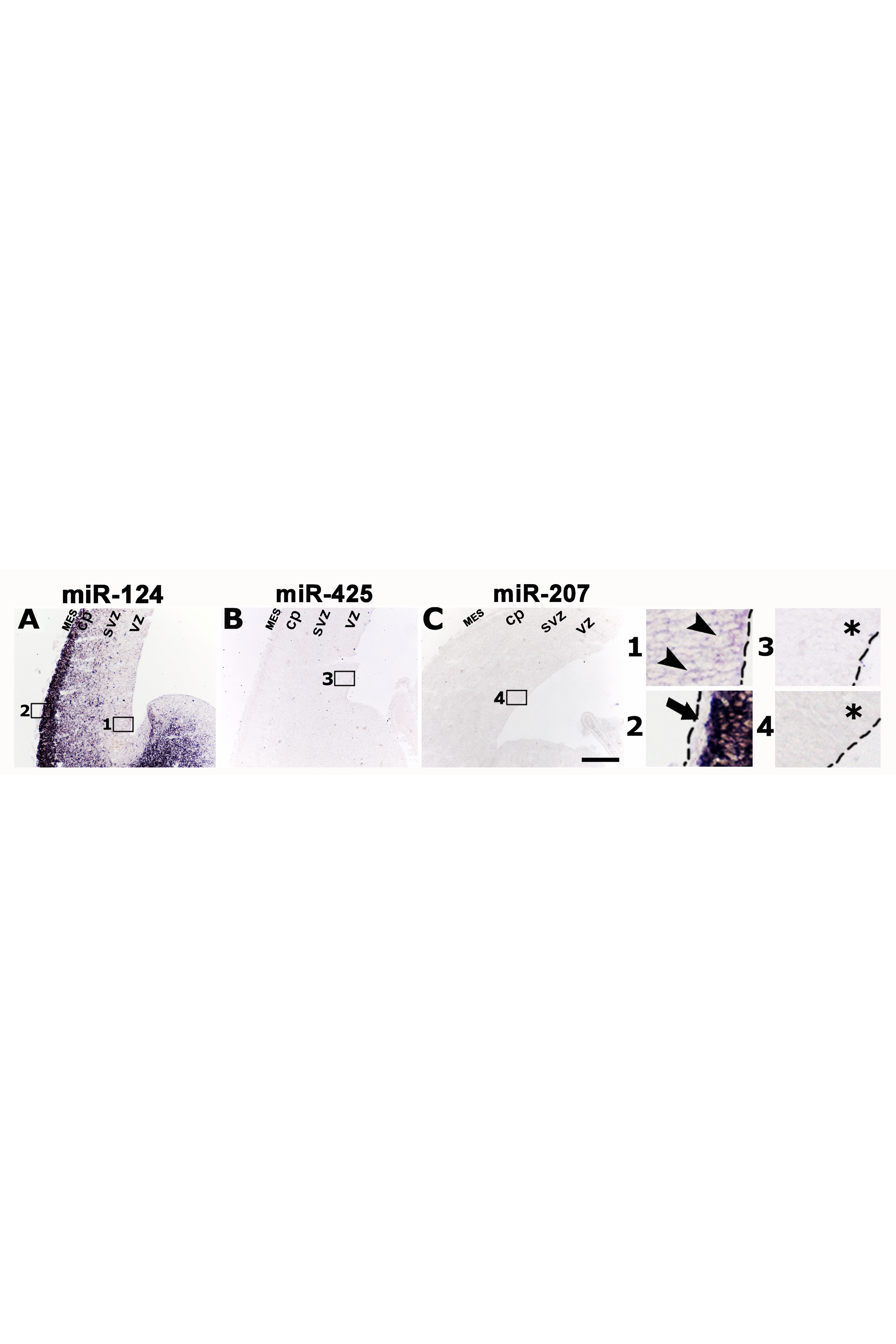

Supplement: Additional file 1 — Specific faint expression of miR-124 in the E14.5 VZ. (A-C) In situ hybridization of miR-124 (A), miR-425 (B) and miR-207 (C) probes on mid-frontal E14.5 telencephalic sections. miR-425 and miR-207 are two miRNAs not expressed in the developing CNS [45,46]. Magnifications of boxed areas illustrate the faint staining detectable in the VZ (black arrowheads) but not in mesenchymal tissue (black harrow) upon miR-124 hybridization, as well as the absence of any signal in samples hybridized with miR-425 or miR-207 (asterisks). Scale bar = 100 μm. Abbreviations: cp, cortical plate; mes, mesenchymal tissue; svz, subventricular zone; vz, ventricular zone. [file 1749-8104-4-40-S1.tiff]

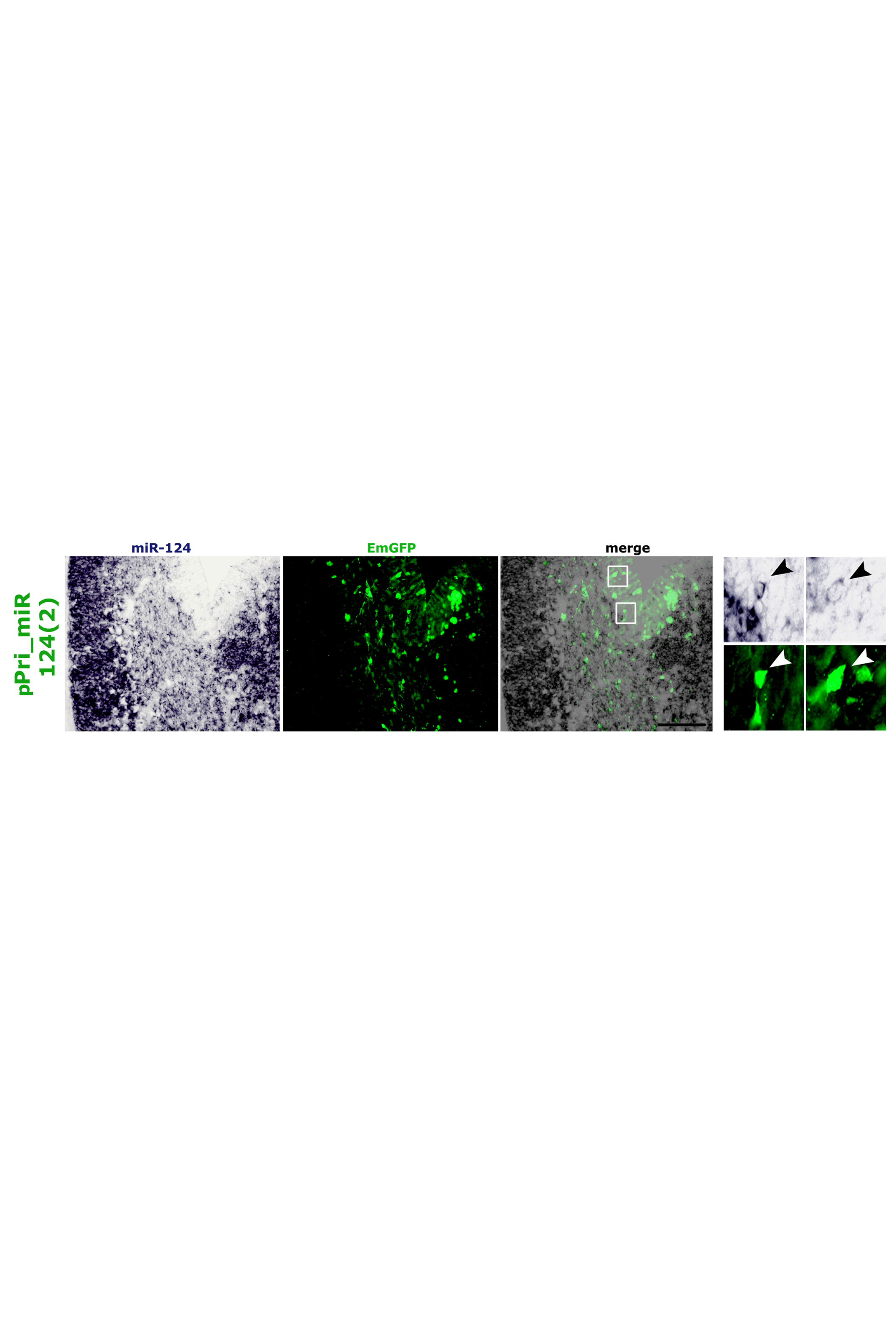

Supplement: Additional file 2 — Levels of miR-124 expression in the E14.5 VZ after in vivo E12.5 pPri-miR-124 electroporation. Arrowheads in boxed inset magnifications denote mid-to-high miR-124 expression levels, which are specifically restricted to heavily electroporated elements. Scale bar = 100 μm. [file 1749-8104-4-40-S2.tiff]

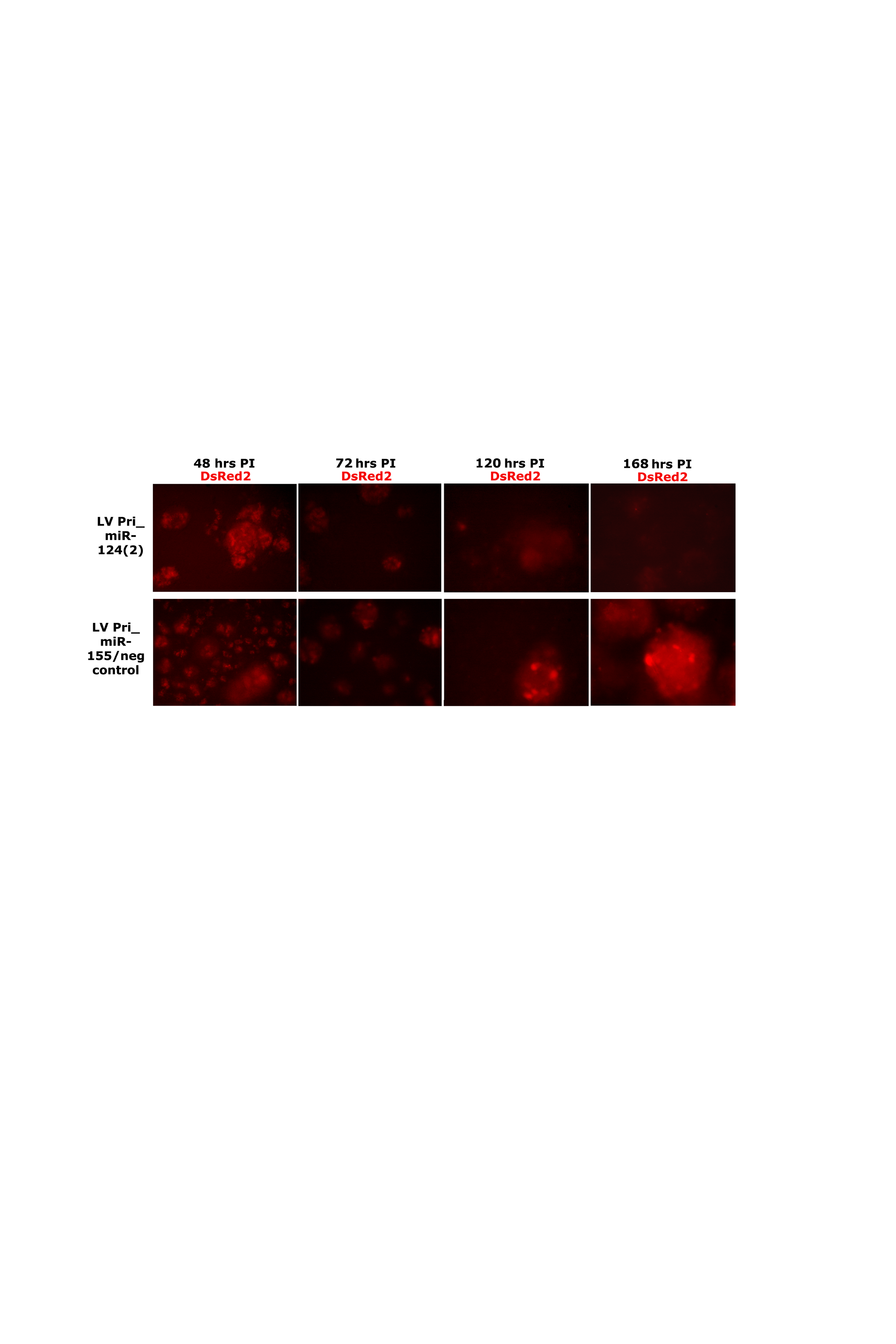

Supplement: Additional file 3 — Time-course DsRed2 fluorescence in primary cortical precursor cultures infected with Pri-miR expressing lentiviruses. Divergent temporal progression of DsRed2 fluorescence in E12.5 neuroblasts infected by LV_Pri-miR-124(2) or LV_Pri-miR-155/neg_control and allowed to differentiate in FCS. PI, post-infection. [file 1749-8104-4-40-S3.tiff]

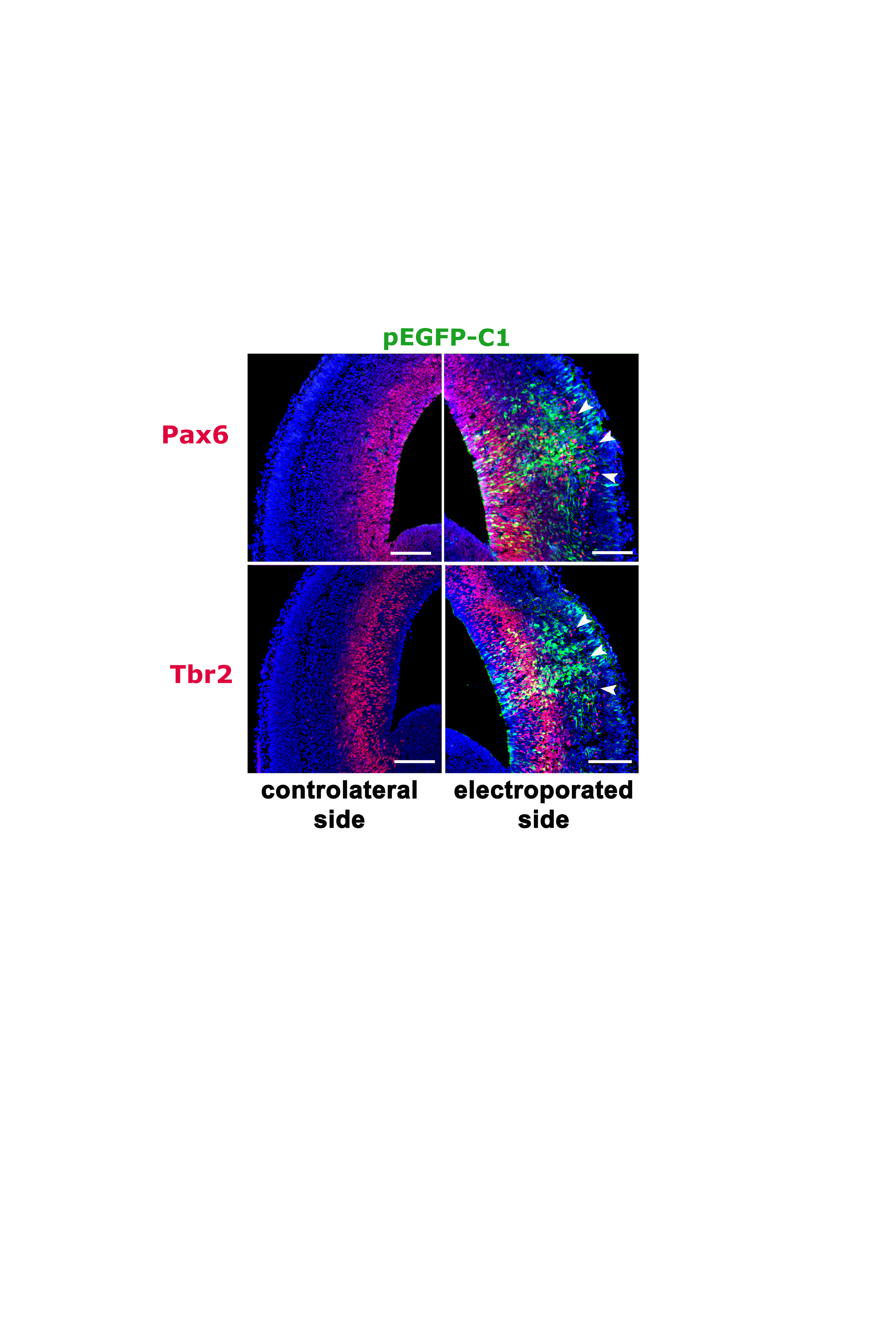

Supplement: Additional file 4 — Displacement of apical, Pax6+, and basal, Tbr2+, precursors within the cortical wall of E14.5 brains electroporated 2 days earlier with pEGFP-C1. Arrowheads point to abventricularly displaced Pax6+ and Tbr2+ elements, both positive and negative for electroporated EGFP. Such displaced cells were not detectable in the controlateral, non-electroporated side of same embryos (N = 3). Scale bar = 100 μm. [file 1749-8104-4-40-S4.tiff]

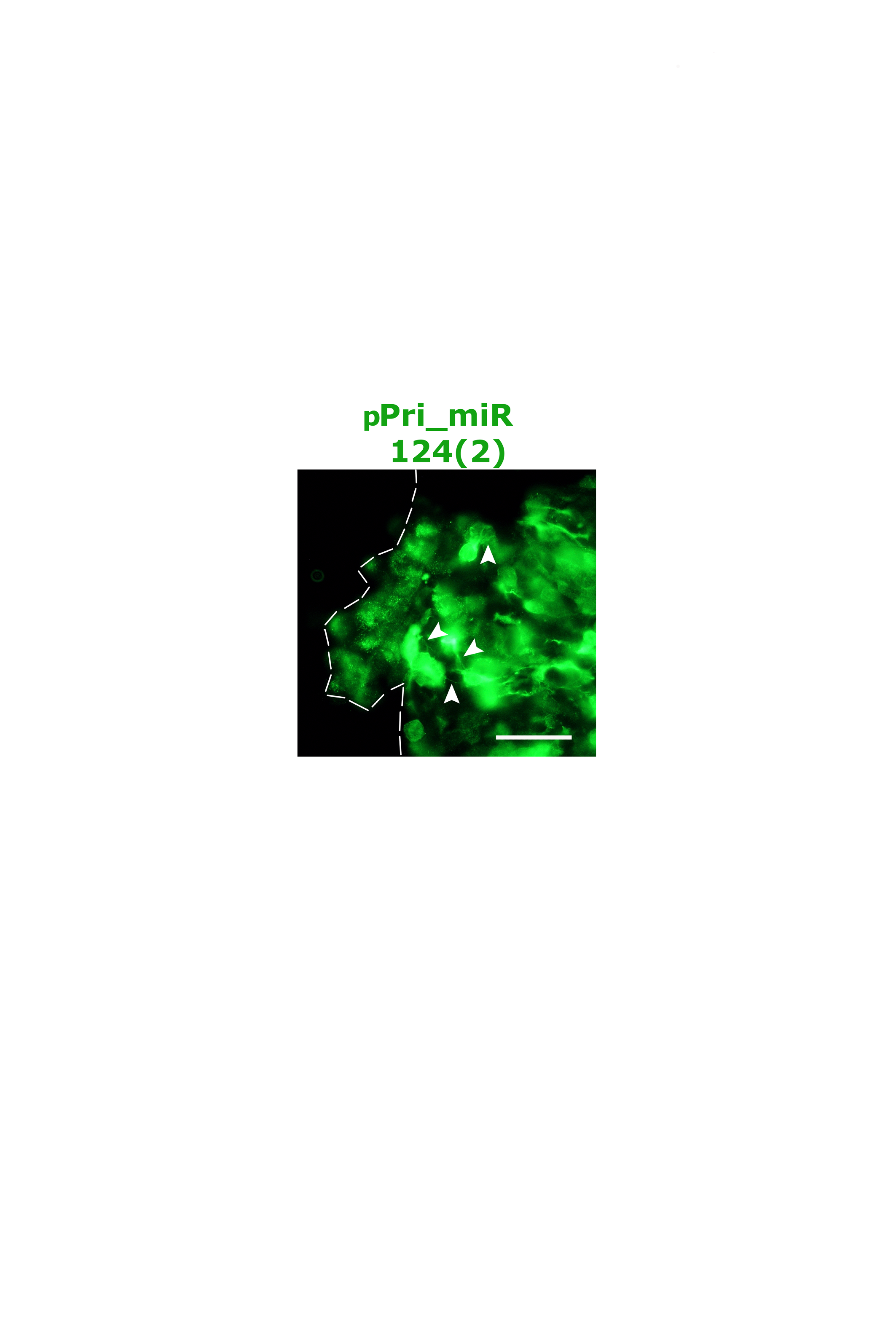

Supplement: Additional file 5 — VZ neuronal differentiation in E14.5 cerebral cortex electroporated 2 days earlier with pPri-miR-124(2). Arrowheads show outgrowing neurites within the VZ. Scale bar = 10 μm. [file 1749-8104-4-40-S5.tiff]

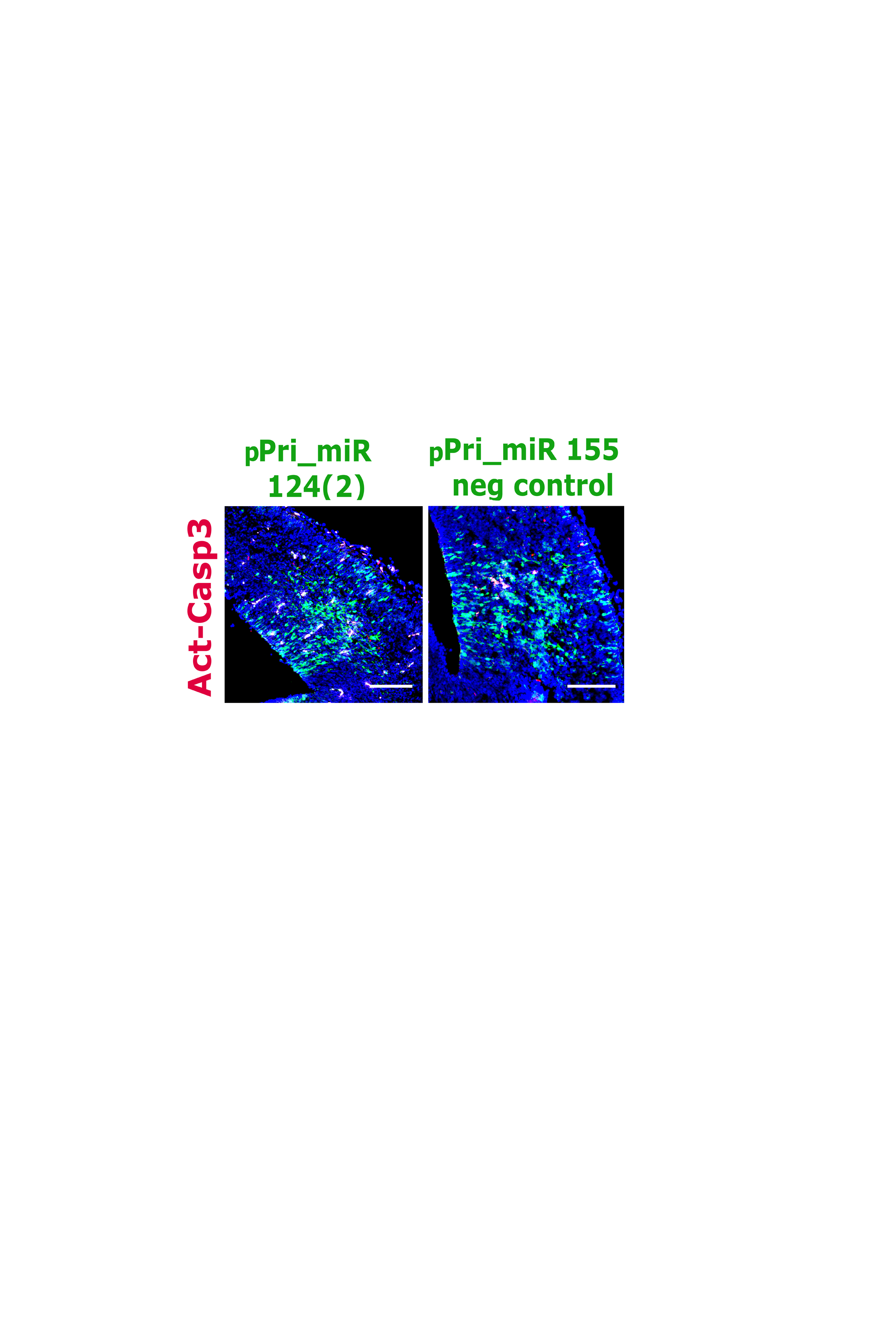

Supplement: Additional file 6 — Distribution of activated-Caspase3+ apoptotic cells within the cortical wall of E14.5 brains electroporated 2 days earlier with pPri-miR-124(2) or pPri-miR-155/neg_control. N = 4+4. Scale bar = 100 μm. [file 1749-8104-4-40-S6.tiff]

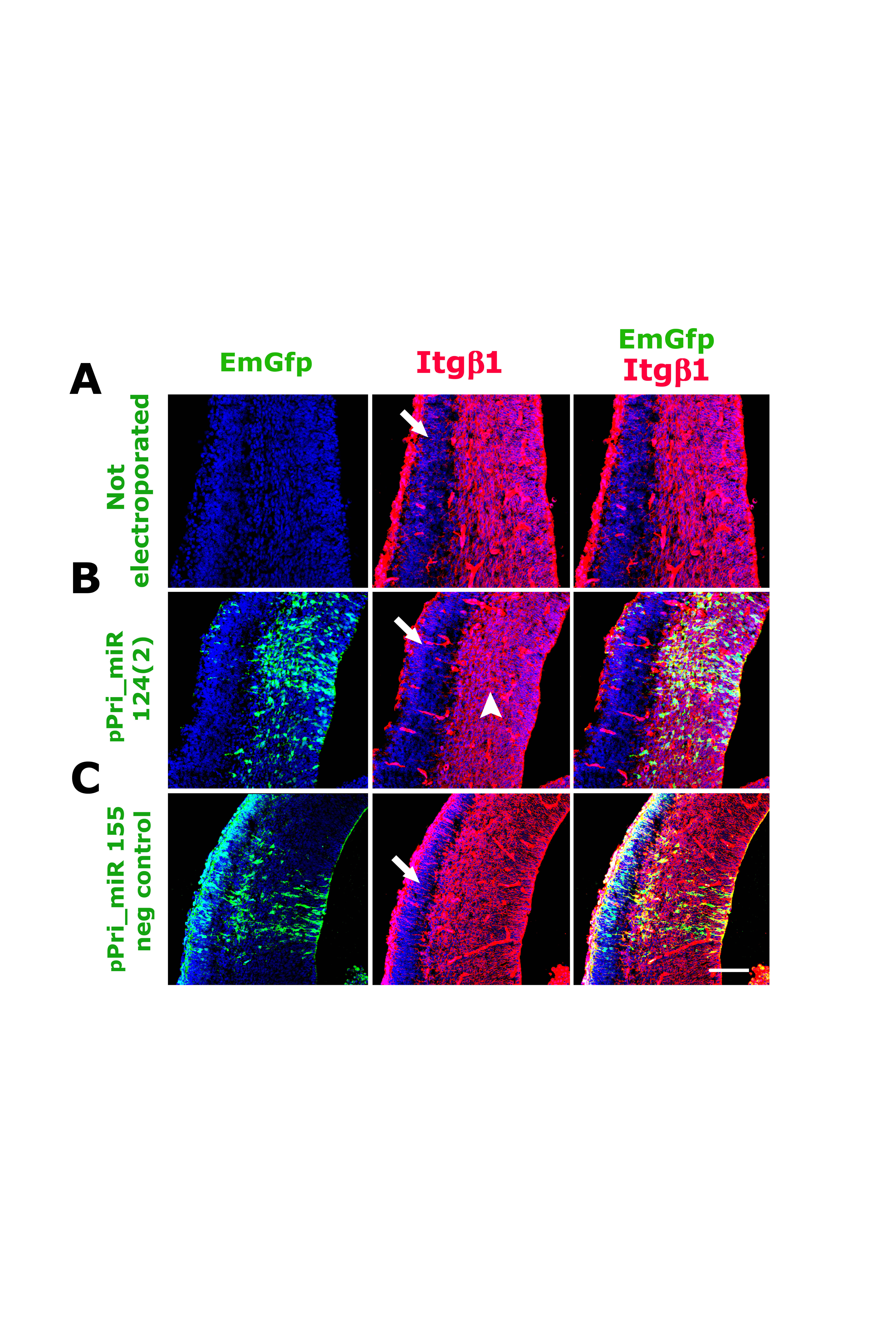

Supplement: Additional file 7 — Distribution of β1-integrin within the cortical wall of E14.5 brains electroporated 2 days earlier with pPri-miR-124(2) or pPri-miR-155/neg_control. The arrowhead in (B) points to the pPri-miR-124(2)-electroporated region, which does not display any overt reduction of β1-integrin immunoreactivity. Arrows in (A-C) denote the cortical plate, where β1-integrin is down-regulated and restricted to radial glial fibers. Scale bar = 100 μm. [file 1749-8104-4-40-S7.tiff]
